# Supplementary material for: Multiomics-based analyses of KPNA2 highlight its multiple potentials in hepatocellular carcinoma
Source: PeerJ. 2021 Sep 21;9:e12197. doi: 10.7717/peerj.12197 (PMC8462373; doi:10.7717/peerj.12197)
Supplement: Supplemental Information 1 — Pre-analysis of KPNA2 in HCC. [file peerj-09-12197-s001.docx]

**Supplementary file 1: Pre-analysis**

**MATERIALS AND METHODS**

**HCC data collection from GEO database and TCGA database**

We screened datasets and series about HCC from GEO database to obtain reliable data for DEGs identification. Eligible chip data were searched with the following strategies: (liver OR hepato*) [Title] AND (cancer OR carcinoma OR tumor OR malignancy) [Title] AND "Homo sapiens" [porgn:__txid9606] (until March 2018). Studies that met the following inclusion criteria were included: 1) the subjects of the experiment were human beings, 2) the study provided gene expression profiling data for 10 and above pairs of HCC and paired adjacent normal liver tissues, 3) the research platform was based on affymetrix human gene or genome arrays, and 4) the studies should be published in English. After the condition filtering, a total of 5 sets of GEO microarray data including GSE60502([Wang et al. 2014](#_ENREF_10)), GSE64041([Makowska et al. 2016](#_ENREF_3)), GSE84402([Hui et al. 2017](#_ENREF_2)), GSE12941([Reiko et al. 2010](#_ENREF_4)), and GSE14520([Roessler et al. 2012](#_ENREF_6); [Stephanie et al. 2010](#_ENREF_9)) were identified from the initial literature and manual search. For GSE14520, two platforms were applied and there were 19 and 213 pairs of samples, respectively (Table 1). In addition, the transcriptome profiling data (counts) of 371 HCC patients from TCGA database (424 samples including 371 primary tumors, 3 recurrent tumors, and 50 paired adjacent normal liver tissues) and their clinical information were downloaded for analysis.

Table 1. Sample data of HCC collected from GEO database.

| Accession/ID | | Platform | Number of paired HCC and adjacent normal tissue samples (pairs) | Number of unpaired HCC and adjacent normal tissue samples^#^ | Pubmed ID |
| --- | --- | --- | --- | --- | --- |
| GSE60502 | GPL96 | | n=18 | 0 | PMID: 25376302 |
| GSE64041 | GPL6244 | | n=60 | 0 | PMID: 27499918 |
| GSE84402 | GPL570 | | n=14 | 0 | PMID: 28810927 |
| GSE12941 | GPL5715 | | n=10 | 0 | PMID: 20388846 |
| GSE14520 | GPL571  GPL3921 | | n=19  n=213 | 5  9 | PMID: 22202459  PMID: 21159642 |

**Identification of effective differential expressed genes (EDEGs)**

For GEO data series, only the paired HCC and adjacent normal liver tissues were included for DEGs identification. The gene expression data were processed with GEO2R (an interactive web tool in GEO database). Statistically significant DEGs were identified with the cut-off criterion adjusted *p* value (adj.*p*) < 0.05 and log2 fold change (∣logFC∣)≥1. For GSE14520, only the genes changed consistently from the two platforms were considered. The TCGA data were processed with EdgeR package in R software([Davis J et al. 2012](#_ENREF_1); [Robinson et al. 2009](#_ENREF_5)). The intersection of the DEGs from the 5 GEO series was validated in TCGA database with the cutoff criterion false discovery rate (FDR)<0.05 and |logFC|≥1. Only the confirmed DEGs were regarded as EDEGs.

**Expression network construction and hub gene selection of the EDEGs**

The Pearson correlations between the EDEGs were analyzed through SPSS 18.0 (SPSS, Chicago, IL, USA). The genes with correlation coefficient (R)≥0.5 and *p*<0.05 were considered to be co-expressed. While those with R≤-0.5 (*p*<0.05) were considered to be negative correlated and they might play opposite roles in some biological processes. The gene expression network was constructed with the co-expressed genes and negatively correlated genes, visualized through Cytoscape 3.2.0([Shannon et al. 2003](#_ENREF_8)). The nodes and lines in the network graphs represented the EDEGs and correlations between them, respectively. The gene centralities were calculated through CentiScape 2.2([Scardoni et al. 2009](#_ENREF_7)) and hub genes were selected with degree≥15.

**Evaluation of prognostic and diagnostic effects of the hub genes**

The univariate Cox regression survival analysis was used to evaluate prognostic power of the hub genes. The multivariate Cox analysis (method: forward stepwise, Likelihood Ratio) was used to find independent prognostic factors with the probability for stepwise entry 0.01 and removal 0.05.

To further investigate the diagnostic value of KPNA2, the receiver operating characteristic (ROC) curve analysis was applied to detect the optimal cut-off point (i.e. that with the highest Youden index which was referred to the sum of sensitivity and specificity minus 1) for separating HCC from normal controls.

**RESULTS**

**Identification of EDEGs in HCC**

After applying cut-off criteria, the up-regulated and down-regulated genes from the 5 GEO data series each were identified. An intersection of 85 DEGs (up-regulated: 22, down-regulated: 63) in HCC were shown (Figure 1).


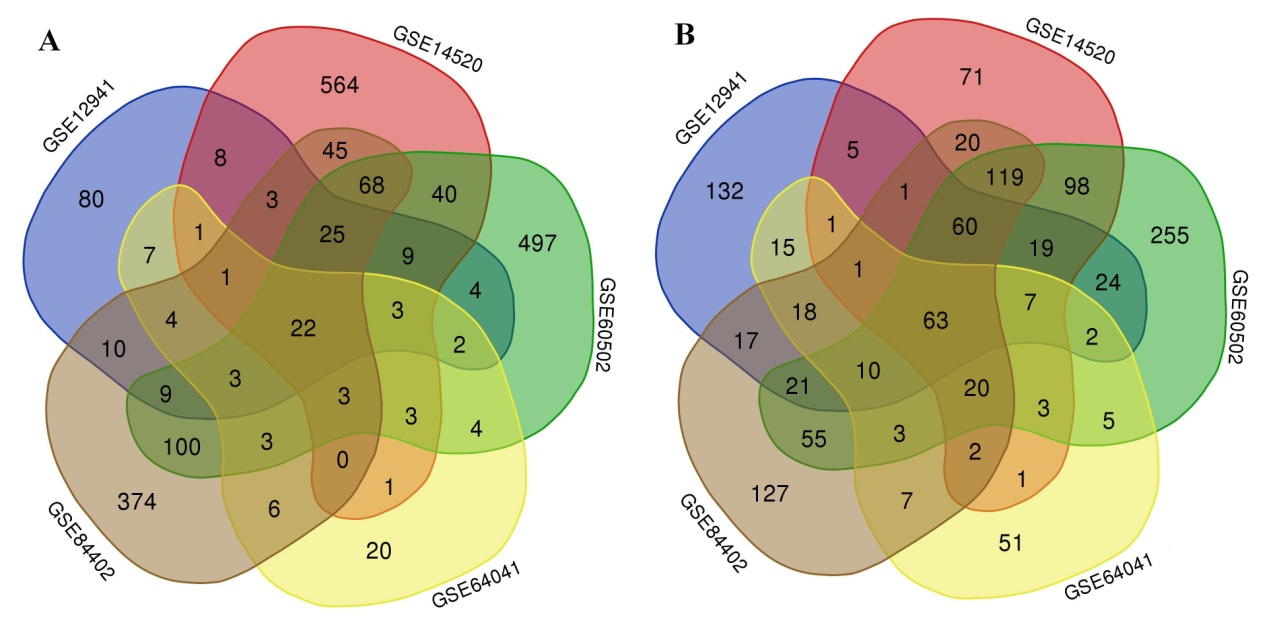


Figure 1. The DEGs of HCC data series from GEO database. A, up-regulated genes; B, down-regulated genes. DEGs, differentially expressed genes.

For TCGA HCC data, with the cut-off criteria, there were 7462 up-regulated and 1565 down-regulated genes in HCC. All the 85 DEGs above were included and all of them were considered EDEGs (Table 2).

Table 2. The expressional differences of the 85 DEGs in HCC from TCGA database

| Up-regulated DEGs(n=22) | | | | Down-regulated DEGs(n=63) | | | | | | | |
| --- | --- | --- | --- | --- | --- | --- | --- | --- | --- | --- | --- |
| Gene | logFC | *P* value | FDR | Gene | logFC | PValue | FDR | Gene | logFC | *P* value | FDR |
| ASPM | 3.746 | 2.06E-46 | 5.78E-44 | ADRA1A | -3.112 | 1.19E-38 | 1.69E-36 | GYS2 | -2.160 | 9.67E-20 | 2.08E-18 |
| AURKA | 2.707 | 7.50E-42 | 1.41E-39 | AKR1D1 | -1.901 | 4.05E-14 | 4.26E-13 | HAO2 | -2.351 | 4.27E-18 | 7.61E-17 |
| CCNB2 | 3.745 | 2.01E-48 | 7.32E-46 | ALDH8A1 | -1.896 | 1.33E-27 | 6.57E-26 | HGF | -2.066 | 1.72E-19 | 3.60E-18 |
| CDK1 | 3.507 | 2.08E-45 | 5.42E-43 | ANK3 | -1.118 | 4.52E-07 | 1.60E-06 | HGFAC | -1.756 | 4.85E-09 | 2.39E-08 |
| CDKN3 | 3.924 | 6.66E-54 | 3.96E-51 | APOF | -2.860 | 1.26E-36 | 1.43E-34 | HPGD | -1.064 | 0.00034 | 0.000735 |
| CENPF | 3.911 | 1.22E-50 | 5.67E-48 | BBOX1 | -1.750 | 8.01E-11 | 5.22E-10 | IGF1 | -1.298 | 7.93E-09 | 3.77E-08 |
| DLGAP5 | 3.721 | 4.28E-45 | 1.08E-42 | C7 | -2.042 | 8.65E-14 | 8.63E-13 | IGFBP3 | -1.976 | 8.91E-37 | 1.02E-34 |
| DTL | 3.283 | 1.57E-40 | 2.73E-38 | C9 | -2.596 | 1.17E-17 | 1.96E-16 | JCHAIN | -1.841 | 2.69E-11 | 1.87E-10 |
| ECT2 | 2.418 | 1.86E-30 | 1.22E-28 | CCL19 | -1.879 | 1.12E-34 | 1.11E-32 | KLKB1 | -1.619 | 7.26E-24 | 2.51E-22 |
| FOXM1 | 3.637 | 3.30E-43 | 6.88E-41 | CD5L | -3.020 | 1.20E-28 | 6.62E-27 | KMO | -1.800 | 3.26E-19 | 6.58E-18 |
| GINS1 | 2.753 | 3.59E-33 | 3.06E-31 | CDHR2 | -1.900 | 8.17E-11 | 5.31E-10 | LPA | -2.330 | 2.06E-29 | 1.22E-27 |
| GPC3 | 5.948 | 7.19E-36 | 7.80E-34 | CETP | -2.811 | 2.16E-64 | 2.18E-61 | LY6E | -1.992 | 3.18E-20 | 7.20E-19 |
| KIF20A | 3.737 | 3.73E-45 | 9.63E-43 | CLEC1B | -5.214 | 1.12E-57 | 8.84E-55 | LYVE1 | -2.978 | 1.92E-51 | 9.66E-49 |
| KPNA2 | 1.446 | 3.18E-24 | 1.14E-22 | CLEC4M | -5.585 | 4.32E-52 | 2.25E-49 | MBL2 | -1.820 | 4.97E-16 | 6.86E-15 |
| MKI67 | 3.382 | 4.94E-40 | 8.23E-38 | CRHBP | -4.476 | 1.37E-78 | 3.34E-75 | MT1G | -2.471 | 2.98E-12 | 2.38E-11 |
| NCAPG | 3.820 | 5.34E-47 | 1.66E-44 | CXCL12 | -2.672 | 8.72E-50 | 3.63E-47 | NAT2 | -2.933 | 8.88E-32 | 6.72E-30 |
| NUSAP1 | 2.282 | 1.94E-32 | 1.57E-30 | CXCL14 | -3.577 | 2.67E-33 | 2.29E-31 | PCK1 | -2.191 | 5.28E-20 | 1.17E-18 |
| PRC1 | 3.248 | 9.30E-49 | 3.43E-46 | CYP2C8 | -2.668 | 1.38E-33 | 1.22E-31 | PDGFRA | -1.577 | 9.89E-10 | 5.43E-09 |
| ROBO1 | 2.922 | 6.90E-26 | 2.89E-24 | CYP39A1 | -2.424 | 3.72E-24 | 1.32E-22 | PLAC8 | -2.295 | 2.43E-25 | 9.64E-24 |
| SQLE | 1.655 | 5.74E-14 | 5.92E-13 | CYP3A43 | -1.412 | 6.75E-08 | 2.75E-07 | PROZ | -1.585 | 3.00E-15 | 3.72E-14 |
| TOP2A | 3.934 | 1.67E-44 | 3.86E-42 | CYP4A11 | -2.225 | 3.73E-29 | 2.15E-27 | RDH16 | -2.012 | 1.88E-18 | 3.50E-17 |
| TPX2 | 2.132 | 1.86E-24 | 6.82E-23 | DCN | -2.309 | 1.70E-21 | 4.48E-20 | SDS | -1.314 | 2.93E-07 | 1.07E-06 |
|  |  |  |  | DNASE1L3 | -2.900 | 1.13E-54 | 7.02E-52 | SLC22A1 | -2.373 | 5.65E-18 | 9.86E-17 |
|  |  |  |  | DPT | -1.998 | 6.63E-12 | 5.03E-11 | SLC38A4 | -1.493 | 1.15E-16 | 1.73E-15 |
|  |  |  |  | F9 | -1.873 | 4.42E-15 | 5.36E-14 | SPP2 | -1.602 | 7.57E-10 | 4.23E-09 |
|  |  |  |  | FBP1 | -2.014 | 3.32E-26 | 1.44E-24 | SRD5A2 | -1.984 | 1.73E-14 | 1.92E-13 |
|  |  |  |  | FCN3 | -4.092 | 2.62E-72 | 4.24E-69 | SRPX | -1.733 | 4.62E-12 | 3.61E-11 |
|  |  |  |  | GBA3 | -2.258 | 5.42E-22 | 1.52E-20 | STAB2 | -4.850 | 3.81E-112 | 5.56E-108 |
|  |  |  |  | GHR | -2.457 | 6.47E-45 | 1.58E-42 | STEAP3 | -1.699 | 1.75E-27 | 8.52E-26 |
|  |  |  |  | GLS2 | -1.957 | 2.14E-11 | 1.51E-10 | TDO2 | -1.791 | 5.65E-15 | 6.75E-14 |
|  |  |  |  | GNMT | -1.483 | 6.82E-09 | 3.28E-08 | VIPR1 | -3.468 | 3.40E-74 | 6.20E-71 |
|  |  |  |  | GPM6A | -3.139 | 4.60E-42 | 8.82E-40 |  |  |  |  |

LogFC, log2-fold change; FDR, false discovery rate.

**Gene expression network construction and hub gene selection**

After removing the isolated nodes, a complex network including 73 nodes (genes) and 504 edges (correlations) was constructed (Figure 2A) and plenty of co-expressions were shown. With the criterion degree>15, 36 hub genes (up-regulated: 19, down-regulated: 17) were identified (Figure 2B). It is interesting that deoxyribonuclease 1-like 3 (DNASE1L3), in addition to its co-expression with other down-regulated genes, was also negatively correlated with 15 up-regulated hub genes.


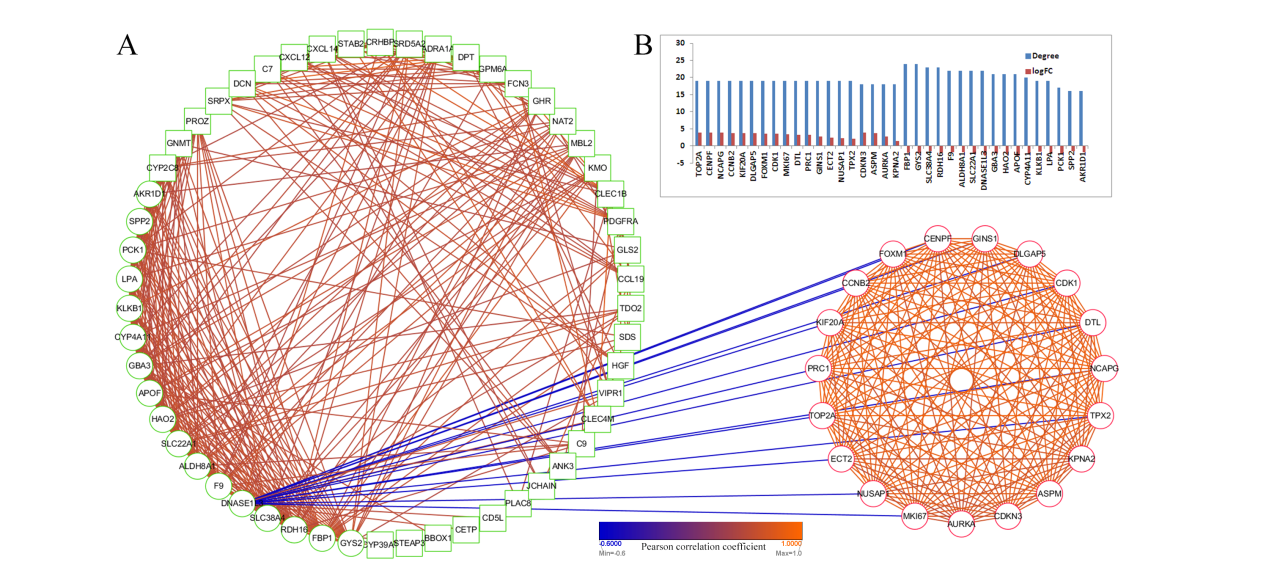


Figure 2. The expression network of the EDEGs and the hub genes. (A) expression network of the EDEGs. Nodes represented the genes (red and green indicated up-regulated and down-regulated genes in HCC, respectively). Lines represented the correlations between the genes (orange indicated co-expressions while blue indicated negative correlations). The nodes with round shape were hub genes. (B) 36 hub genes with degree≥15. Pearson correlation analysis was used and only correlations with │correlation coefficient│≥0.5 were selected for network construction.

**Survival analysis of the hub genes in HCC patients**

Through univariate Cox regression analysis, except GBA3, 35 of the 36 hub genes were indicated to be prognostic factors for HCC survival (*p*<0.05) (Table 3). Through multivariate Cox regression analysis, with age at diagnosis, pathological grade, pathological stage, and the 35 genes above, KPNA2 and pathological stage, were found to be unfavorable independent prognostic factors with hazard ratio (HR) 1.657 (95%CI: 1.333-2.060) and 1.503 (95%CI: 1.212-1.865), respectively (Table 4). Their prognostic power was also visualized through Kaplan-Meier survival analyses (Figure 3A-B).

Table 3 Results of univariate Cox regression analysis of hub genes for LIHC survival

| Up-regulated hub genes (n=19) | | | Down-regulated hub genes (n=17) | | |
| --- | --- | --- | --- | --- | --- |
| Gene | HR (95.0% CI) | P value |  | HR (95.0% CI) | P value |
| KPNA2 | 1.803 (1.472-2.209) | 1.246E-08** | DNASE1L3 | 0.834 (0.768-0.905) | 1.468E-05** |
| TPX2 | 1.415 (1.229-1.630) | 1.399E-06** | KLKB1 | 0.852 (0.770-0.942) | 0.002** |
| KIF20A | 1.386 (1.213-1.584) | 1.610E-06** | FBP1 | 0.887 (0.817-0.963) | 0.004** |
| DLGAP5 | 1.350 (1.1893-1.532) | 3.360E-06** | ALDH8A1 | 0.894 (0.831-0.962) | 0.003** |
| ECT2 | 1.344 (1.170-1.543) | 2.806E-05** | RDH16 | 0.895 (0.844-0.950) | 2.651E-04** |
| GINS1 | 1.324(1.160-1.510) | 2.907E-05** | SLC22A1 | 0.910 (0.865-0.957) | 2.513E-04** |
| NCAPG | 1.321 (1.159-1.505) | 2.911E-05** | LPA | 0.910 (0.853-0.971) | 0.004** |
| CDK1 | 1.297 (1.142-1.473) | 6.230E-05** | SPP2 | 0.912 (0.870-0.956) | 1.159E-4** |
| MKI67 | 1.280 (1.132-1.449) | 8.846E-05** | SLC38A4 | 0.915 (0.855-0.980) | 0.011* |
| PRC1 | 1.262 (1.100-1.447) | 9.068E-04** | AKR1D1 | 0.920 (0.872-0.971) | 0.002** |
| FOXM1 | 1.248 (1.109-1.403) | 2.326E-04** | APOF | 0.926 (0.875-0.981) | 0.009** |
| CENPF | 1.236 (1.097-1.394) | 5.186E-04** | PCK1 | 0.928 (0.877-0.981) | 0.008** |
| TOP2A | 1.228 (1.098-1.373) | 3.204E-04** | CYP4A11 | 0.928 (0.870-0.989) | 0.022* |
| CCNB2 | 1.221 (1.078-1.382) | 1.621E-03** | GYS2 | 0.936 (0.890-0.985) | 0.010* |
| ASPM | 1.220 (1.081-1.375) | 1.212E-03** | F9 | 0.943 (0.897-0.992) | 0.023* |
| AURKA | 1.216 (1.054-1.404) | 7.386E-03** | HAO2 | 0.944 (0.896-0.994) | 0.029* |
| CDKN3 | 1.207 (1.069-1.363) | 2.410E-03** | GBA3 | 0.960 (0.907-1.016) | 0.158 |
| DTL | 1.203 (1.064-1.361) | 3.196E-03** |  |  |  |
| NUSAP1 | 1.174 (1.017-1.355) | 2.873E-02* |  |  |  |

HR, hazard ratio; CI, confidence interval. *, *p* < 0.05; **, *p* < 0.001; *p*<0.05 was considered to be statistically significant.

Table 4. Forward stepwise Cox regression (Likelihood Ratio) analysis of the hub genes in HCC survival

|  | B | SE | HR (95.0% CI) | P |
| --- | --- | --- | --- | --- |
| KPNA2 | 0.505 | 0.111 | 1.657 (1.333-2.060) | 5.269E-6** |
| Pathological stage | 0.408 | 0.110 | 1.503 (1.212-1.865) | 2.130E-4** |


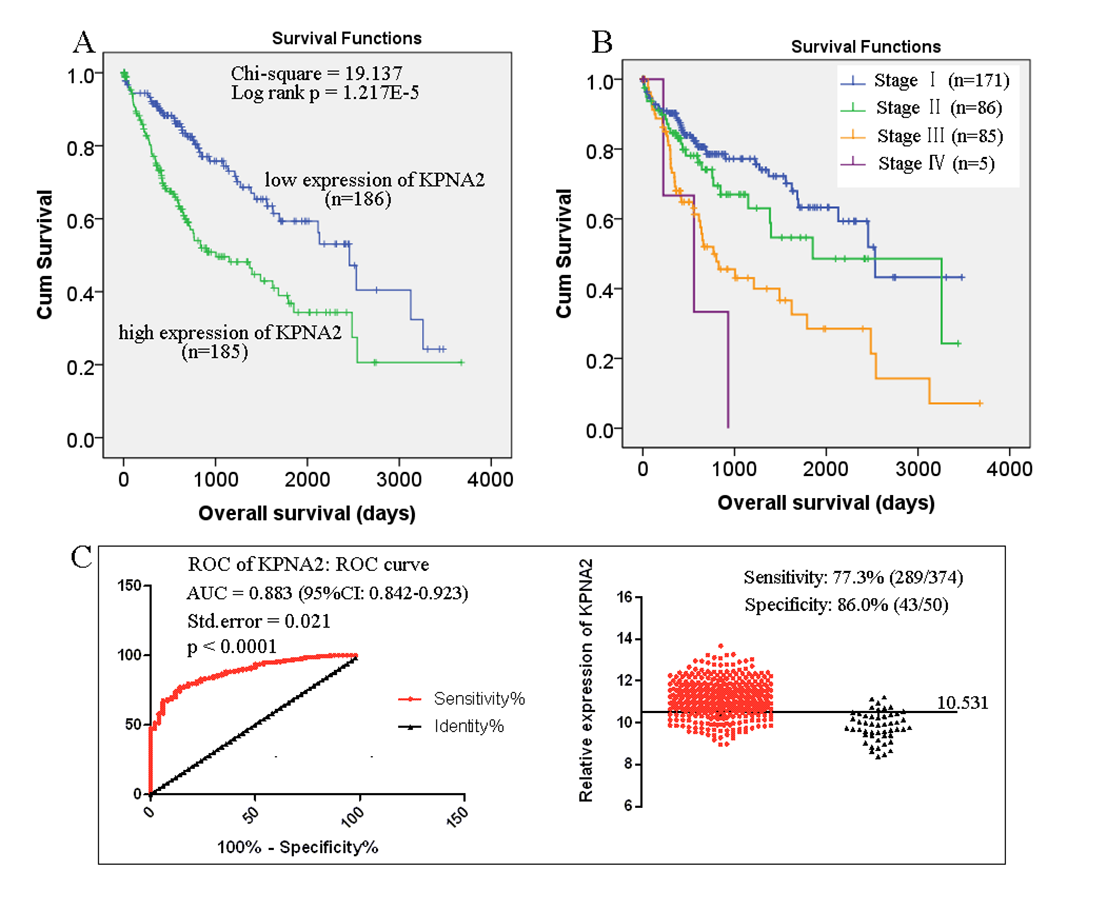


Figure 3. Prognostic and diagnostic power of KPNA2. (A), HCC patients with higher expression of KPNA2 have a poorer overall survival than those with lower expression; Kaplan-meier survival analysis was used and the median gene expression of KPNA was set as threshold for grouping. (B), pathological stage was an independent unfavorable prognostic factor overall survival of HCC patients; (C), ROC curve analysis indicates the good diagnostic power of KPNA2 in gene expression level for HCC. With an optical cutoff value, the sensitivity and specificity were 77.3% and 86.0%, respectively. ROC, receiver operating characteristic. For all the analysis above, *p*<0.05 was considered to be statistically significant.

As KPNA2 had its prognostic values in HCC patients of stage I, stage II, and stage III+IV, individually, the independent prognostic role of KPNA2 was confirmed (Table 5).

Table 5. The prognostic power of KPNA2 in HCC patients of different stages in HCC survival

| HCC patients | B | SE | HR(95.0% CI) | P |
| --- | --- | --- | --- | --- |
| Stage I (n=171) | 0.629 | 0.207 | 1.875 (1.251-2.811) | 0.002* |
| Stage II (n=86) | 0.491 | 0.232 | 1.634(1.037-2.573) | 0.034* |
| Stage III+IV (n=90) | 0.511 | 0.167 | 1.667(1.201-2.314) | 0.002* |

Univariate Cox analysis was used and *p*<0.05 was considered to be statistically significant. B, regression coefficient. SE, standard error. HR, hazard ratio. CI, confidence interval. *, *p* < 0.05;

**The diagnostic power of KPNA2 for HCC in gene expression level**

Through ROC curve analysis (Figures 3C), when discriminating HCC from normal liver tissues, the area under the curve (AUC) of KPNA2 was 0.883 (95%CI, 0.842-0.923) (*p*<0.001), indicating the diagnostic value of KPNA2 in gene expression level.

**REFERENCES**

Davis J M, Yunshun C, and Gordon K S. 2012. Differential expression analysis of multifactor RNA-Seq experiments with respect to biological variation. 40:4288-4297.

Hui W, Huo X, Yang XR, Jia H, Cheng L, Na W, Xuan D, Jin H, Ning W, and Wang C. 2017. STAT3-mediated upregulation of lncRNA HOXD-AS1 as a ceRNA facilitates liver cancer metastasis by regulating SOX4. *Molecular Cancer* 16:136.

Makowska Z, Boldanova T, Adametz D, Quagliata L, Vogt JE, Dill MT, Matter MS, Roth V, Terracciano L, and Heim MH. 2016. Gene expression analysis of biopsy samples reveals critical limitations of transcriptome-based molecular classifications of hepatocellular carcinoma. *Journal of Pathology Clinical Research* 2:80-92.

Reiko S, Miki S, Yae K, Fumitaka T, Hidenori O, Takafumi J, Kazufumi H, Tomoo K, Takahiro O, and Setsuo H. 2010. Combined functional genome survey of therapeutic targets for hepatocellular carcinoma. *Clinical Cancer Research* 16:2518-2528.

Robinson MD, McCarthy DJ, and Smyth GK. 2009. edgeR: a Bioconductor package for differential expression analysis of digital gene expression data. *Bioinformatics* 26:139-140. 10.1093/bioinformatics/btp616

Roessler S, Long EL, Budhu A, Chen Y, Zhao X, Ji J, Walker R, Jia HL, Ye QH, and Qin LX. 2012. Integrative Genomic Identification of Genes on 8p Associated With Hepatocellular Carcinoma Progression and Patient Survival. *Gastroenterology* 142:957-966.e912.

Scardoni G, Petterlini M, and Laudanna C. 2009. Analyzing biological network parameters with CentiScaPe. *Bioinformatics* 25:2857-2859. 10.1093/bioinformatics/btp517

Shannon P, Markiel A, Ozier O, Baliga NS, Wang JT, Ramage D, Amin N, Schwikowski B, and Ideker T. 2003. Cytoscape: a software environment for integrated models of biomolecular interaction networks. *Genome Res* 13:2498-2504. 10.1101/gr.1239303

Stephanie R, Hu-Liang J, Anuradha B, Marshonna F, Qing-Hai Y, Ju-Seog L, Thorgeirsson SS, Zhongtang S, Zhao-You T, and Lun-Xiu Q. 2010. A unique metastasis gene signature enables prediction of tumor relapse in early-stage hepatocellular carcinoma patients. *Cancer Research* 70:10202-10212.

Wang YH, Cheng TY, Chen TY, Chang KM, Chuang VP, and Kao KJ. 2014. Plasmalemmal Vesicle Associated Protein (PLVAP) as a therapeutic target for treatment of hepatocellular carcinoma. *BMC Cancer* 14:815.
