# Supplementary material for: Multiomics-based analyses of KPNA2 highlight its multiple potentials in hepatocellular carcinoma
Source: PeerJ. 2021 Sep 21;9:e12197. doi: 10.7717/peerj.12197 (PMC8462373; doi:10.7717/peerj.12197)
Supplement: Supplemental Information 2 [file peerj-09-12197-s002.docx]

**Supplementary files**

**Table S1.** Clinical features of the plasma samples in ELISA analysis.

| Clinical features | HCC(n=51) | Normal(n=41) | *P* value |
| --- | --- | --- | --- |
| Age (y) |  |  |  |
| < 60 / ≥60 | 38/13 | 28/13 | 0.671 |
| Gender |  |  |  |
| Male/female | 42/9 | 29/12 | 0.285 |
| Etiology |  |  |  |
| HBV/HCV/alcohol/unknown | 37/5/4/5 |  |  |
| TNM stage |  |  |  |
| Ⅰ/Ⅱ/Ⅲ/Ⅳ | 11/8/22/10 |  |  |
| Pathological grade |  |  |  |
| G1/G2/G3 | 9/22/20 |  |  |

**Table S2**. Correlations of KPNA2 methylation and KPNA2 expression in HCC

|  | Distance_closest_TSS (bp） | Correlation | P value |
| --- | --- | --- | --- |
| cg23206777 | -399 | -0.210 | 4.734E-05^**^ |
| cg21018429 | -171 | -0.183 | 3.834E-04^**^ |
| cg21820889 | -102 | -0.159 | 0.002^**^ |
| cg22429852 | -1294 | -0.133 | 0.010^*^ |
| cg06390411 | -42 | -0.063 | 0.228 |
| cg14898140 | 24 | -0.048 | 0.352 |
| cg13777502 | -33 | -0.047 | 0.369 |
| cg16501440 | -6 | -0.045 | 0.383 |
| cg14732136 | -19 | -0.019 | 0.714 |
| cg09570855 | 18312 | -0.015 | 0.766 |
| cg17985418 | -62 | 0.052 | 0.318 |
| cg05771398 | -17 | 0.055 | 0.291 |
| cg09771049 | -49 | 0.072 | 0.168 |

^**^, *p* < 0.01; ^*^, *p* < 0.05. TSS, transcript start site. Spearman correlation analysis was used and *p* < 0.05 was considered significant.

**Table S3**. Age, gender and tumor stage- corrected prognostic effects of KPNA2 CNV and KPNA2 methylation in HCC overall survival.

| Variable | HR (95%CI) | P value |
| --- | --- | --- |
| KPNA2 CNV | 1.73 (0.905-3.32) | 0.0971 |
| cg06390411 | 2.9e+07 (9.85e-08-8.52e+21) | 0.312 |
| cg21820889 | 3.6e-13 (8.81e-28-147) | 0.0951 |
| cg22429852 | 1.16 (0.354-3.79) | 0.807 |
| cg14898140 | 1770 (0.0354-88400000) | 0.176 |
| cg05771398 | 5.38e+09 (0.0772-3.75e+20) | 0.0786 |
| cg09771049 | 17.4 (0.0232-13100) | 0.398 |
| cg16501440 | 12700 (0.00598-2.71e+10) | 0.204 |
| cg13777502 | 71.7 (0.797-6440) | 0.0627 |
| cg09570855 | 0.556 (0.157-1.97) | 0.364 |
| cg14732136 | 5700 (0.00377-8.64e+09) | 0.234 |
| cg21018429 | 3.79e-27 (1.1e-57-13000) | 0.0899 |
| cg23206777 | 0.000138 (5.5e-08-0.346) | 0.0261^*^ |
| cg17985418 | 277 (13.6-5670) | 0.000258^**^ |

^**^, *p* < 0.01; ^*^, *p* < 0.05. Cox regression analysis was used and *p* < 0.05 was considered significant.

**Table S4**. Age, gender and tumor stage- corrected prognostic effects of KPNA2 CNV and KPNA2 methylation in HCC disease-free survival.

| Variable | HR (95%CI) | P value |
| --- | --- | --- |
| KPNA2 CNV | 2.47 (1.39-4.36) | 0.00191^**^ |
| cg06390411 | 7.77e+10 (0.585-1.03e+22) | 0.055 |
| cg21820889 | 1.91e-07 (1.46e-19-250000) | 0.277 |
| cg22429852 | 0.661 (0.243-1.8) | 0.417 |
| cg14898140 | 229000 (33.7-1.56e+09) | 0.00612^**^ |
| cg05771398 | 0.24 (1.26e-10-4.58e+08) | 0.896 |
| cg09771049 | 0.144 (0.000568-36.4) | 0.492 |
| cg16501440 | 0.0458 (1.04e-07-20300) | 0.642 |
| cg13777502 | 30 (0.758-1190) | 0.0699 |
| cg09570855 | 1.32 (0.474-3.66) | 0.597 |
| cg14732136 | 0.109 (8.65e-08-137000) | 0.757 |
| cg21018429 | 86400000 (1.15e-09-6.49e+24) | 0.357 |
| cg23206777 | 0.00329 (9.14e-06-1.18) | 0.0568 |
| cg17985418 | 13.7 (0.0818-2290) | 0.316 |

^**^, *p* < 0.01; ^*^, *p* < 0.05. Cox regression analysis was used and *p* < 0.05 was considered significant.

**Table S5.** The correlations of KPNA2 expression with the fatty acid metabolism pathway genes in HCC.

| Fatty acid metabolism pathway gene | Spearman correlation | *P* value |
| --- | --- | --- |
| ALDH2 | -0.49236 | 0^**^ |
| ADH1B | -0.45806 | 0^**^ |
| ACAT1 | -0.45264 | 0^**^ |
| CYP4A11 | -0.43832 | 0^**^ |
| EHHADH | -0.43682 | 0^**^ |
| CYP4A22 | -0.43437 | 0^**^ |
| ADH4 | -0.42961 | 0^**^ |
| ADH1C | -0.4234 | 0^**^ |
| ACADVL | -0.39725 | 1.78E-15^**^ |
| ECHS1 | -0.38719 | 1.02E-14^**^ |
| ACADS | -0.38698 | 1.07E-14^**^ |
| GCDH | -0.38271 | 2.18E-14^**^ |
| HADH | -0.37718 | 5.46E-14^**^ |
| CPT2 | -0.35487 | 1.89E-12^**^ |
| HADHB | -0.35333 | 2.38E-12^**^ |
| ADH6 | -0.34961 | 4.17E-12^**^ |
| ACAA1 | -0.34162 | 1.36E-11^**^ |
| ACADL | -0.33483 | 3.60E-11^**^ |
| ADH1A | -0.33344 | 4.39E-11^**^ |
| ECI2 | -0.32011 | 2.76E-10^**^ |
| ECI1 | -0.31671 | 4.35E-10^**^ |
| ACADM | -0.31209 | 7.99E-10^**^ |
| ACADSB | -0.30747 | 1.45E-09^**^ |
| ALDH9A1 | -0.30412 | 2.23E-09^**^ |
| ACOX1 | -0.30124 | 3.20E-09^**^ |
| ACAA2 | -0.29127 | 1.09E-08^**^ |
| ACSL1 | -0.28664 | 1.90E-08^**^ |
| ALDH7A1 | -0.26317 | 2.71E-07^**^ |
| ALDH3A2 | -0.25769 | 4.85E-07^**^ |
| ADH5 | -0.25014 | 1.06E-06^**^ |
| CPT1A | -0.24696 | 1.47E-06^**^ |
| ADH7 | -0.22251 | 1.52E-05^**^ |
| ACSL5 | -0.2193 | 2.03E-05^**^ |
| ALDH1B1 | -0.21805 | 2.27E-05^**^ |
| CPT1C | -0.18355 | 0.00038^**^ |
| ACSL6 | -0.1773 | 0.000602^**^ |
| HADHA | -0.1269 | 0.014448^*^ |
| ACOX3 | -0.09193 | 0.076973 |
| CPT1B | -0.05787 | 0.266231 |
| ACAT2 | 0.135061 | 0.009197^**^ |
| ACSL4 | 0.158153 | 0.002249^**^ |
| ACSL3 | 0.214108 | 3.20E-05^**^ |

^*^, *p* <0.05; ^**^, *p* < 0.01. Spearman correlation analysis was used and p < 0.05 was considered significant.

Table S6. The correlations of KPNA2 transcripts with ALF and ALB expressions in HCC.

|  | AFP | |  | ALB | |
| --- | --- | --- | --- | --- | --- |
|  | Correlation coefficient | P value |  | Correlation coefficient | P value |
| ENST00000330459 | 0.223 | 1.583E-05** |  | -0.280 | 4.384E-08** |
| ENST00000537025 | -0.014 | 0.787 |  | -0.063 | 0.228 |
| ENST00000583392 | 0.052 | 0.321 |  | -0.147 | 0.005** |
| ENST00000582898 | 0.189 | 2.538E-04** |  | -0.083 | 0.110 |

*, *p*<0.05, **, *p*<0.01, Spearman correlation analysis was used and p<0.05 was considered significant.

**Figure S1**


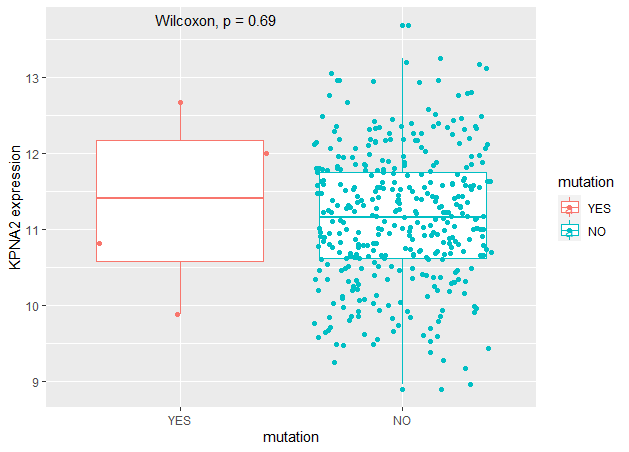


**Figure S1.** The effects of KPNA2 mutation on KPNA2 expression. Wilcoxon test was used for the comparison of KPNA2 expression between the two groups and p<0.05 was considered significant.

**Figure S2**


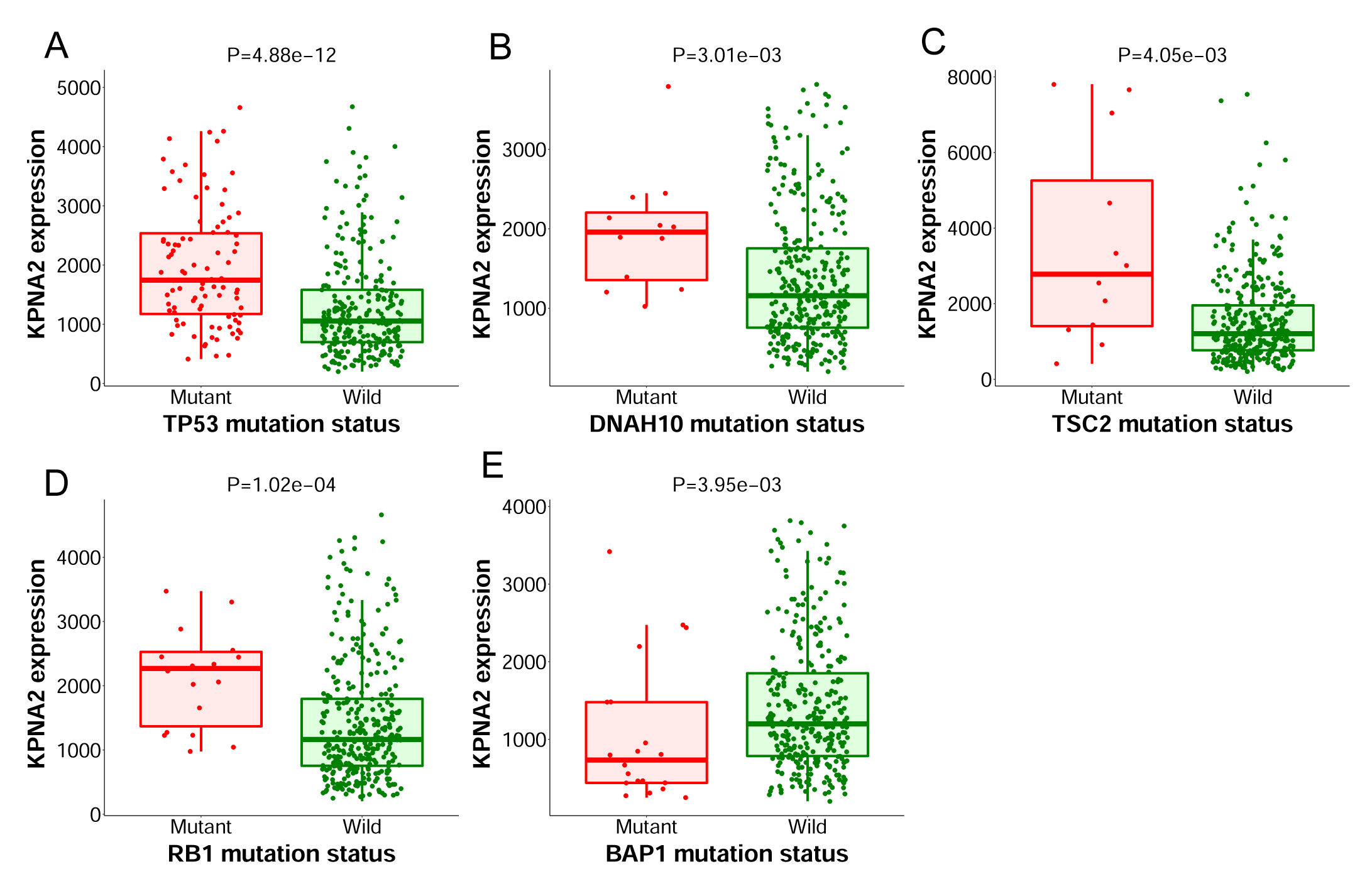


**Figure S2.** Mutations of other genes and KPNA2 expression in TCGA-HCC samples. (A-D) Comparing with the wild type HCC samples, KPNA2 was shown to be higher expressed in HCC samples with TP53, DNAH10, TSC, and RB1 mutations, respectively. (E) BAP1mutation was shown to have a negative effect on KPNA2 expression in HCC samples. Wilcoxon test was used for the analysis and *p* < 0.05 was considered significant.

**Figure S3**

**Figure S3.** ROC curve analysis of KPNA2 expression in discriminating of the OS status of HCC patients. For the analysis, timeROC package in R was used and the 1-year, 3-year and 5-year AUCs were shown.

**Figure S4**

**
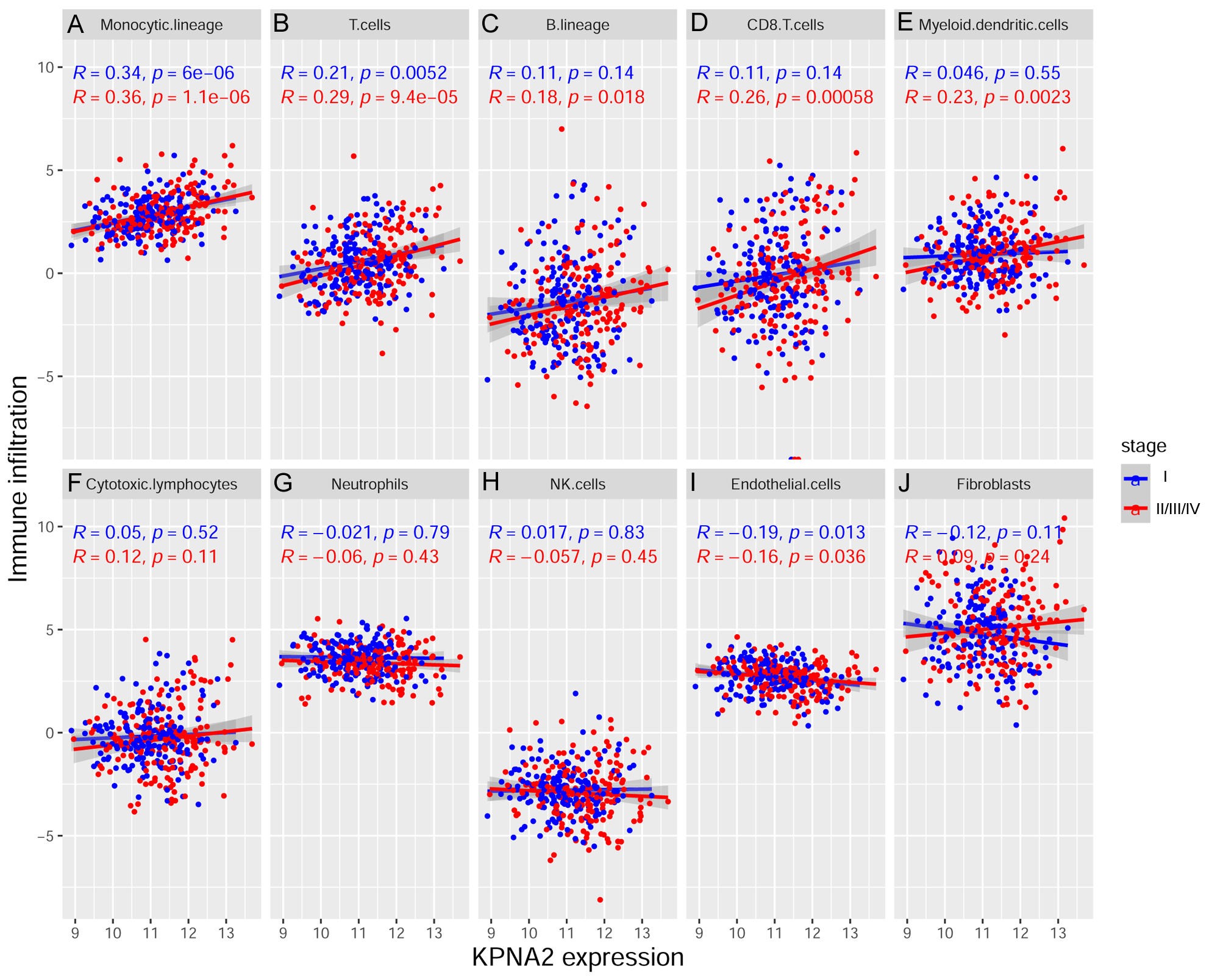
**

**Figure S4.** The correlations of KPNA2 with immune infiltrations in HCC samples of different stages. Spearman correlation analysis was used and *p* < 0.05 was considered significant.

**Figure S5**


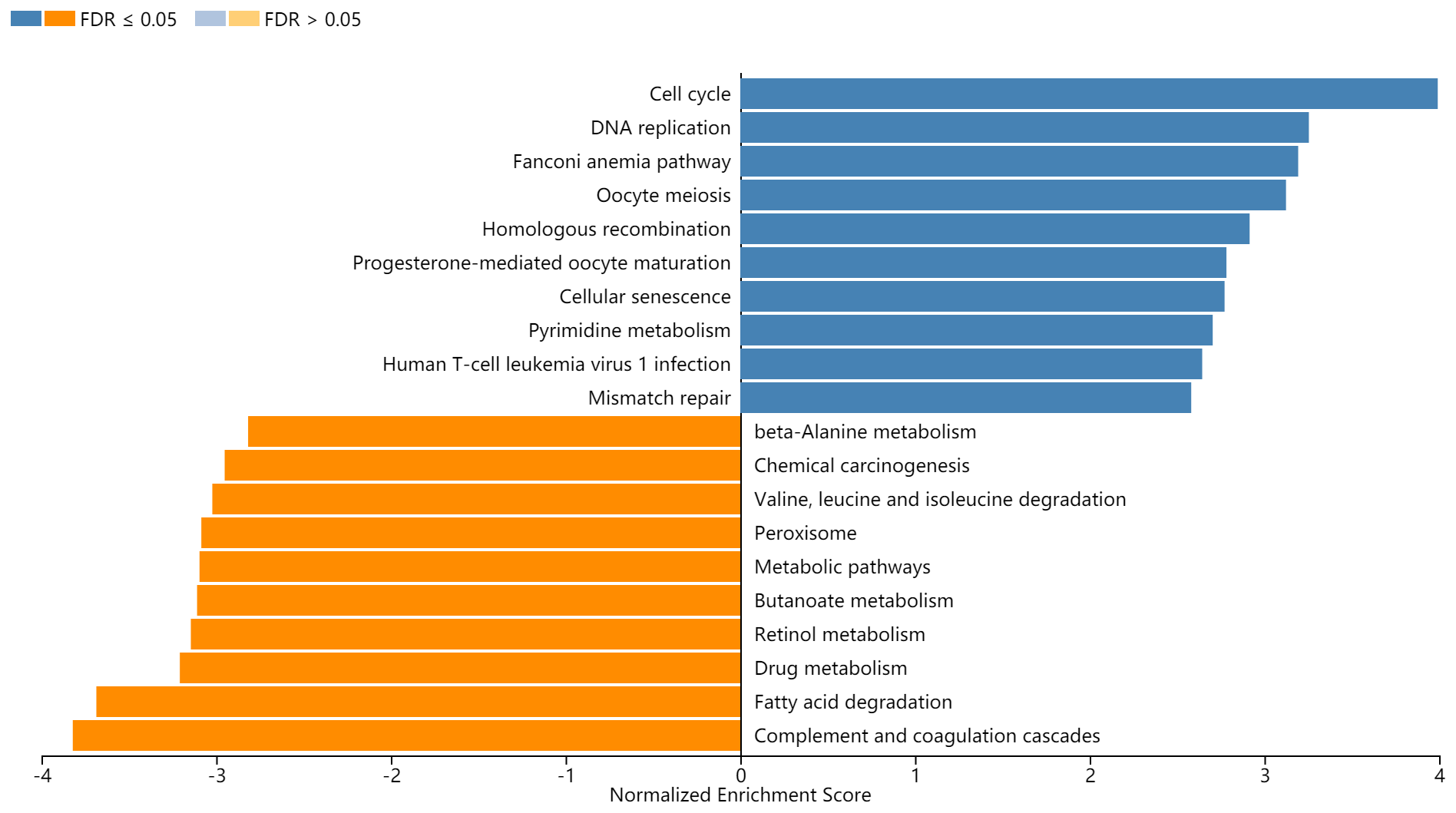


**Figure S5.** Gene set enrichment analysis (GSEA) of KPNA2-correlated genes in HCC. The GSEA was perform with WEB-based GEne SeT AnaLysis Toolkit ([http://www.webgestalt.org/#](http://www.webgestalt.org/)) and FDR < 0.05 was considered significant. FDR, false discovery rate.

**Figure S6**

**
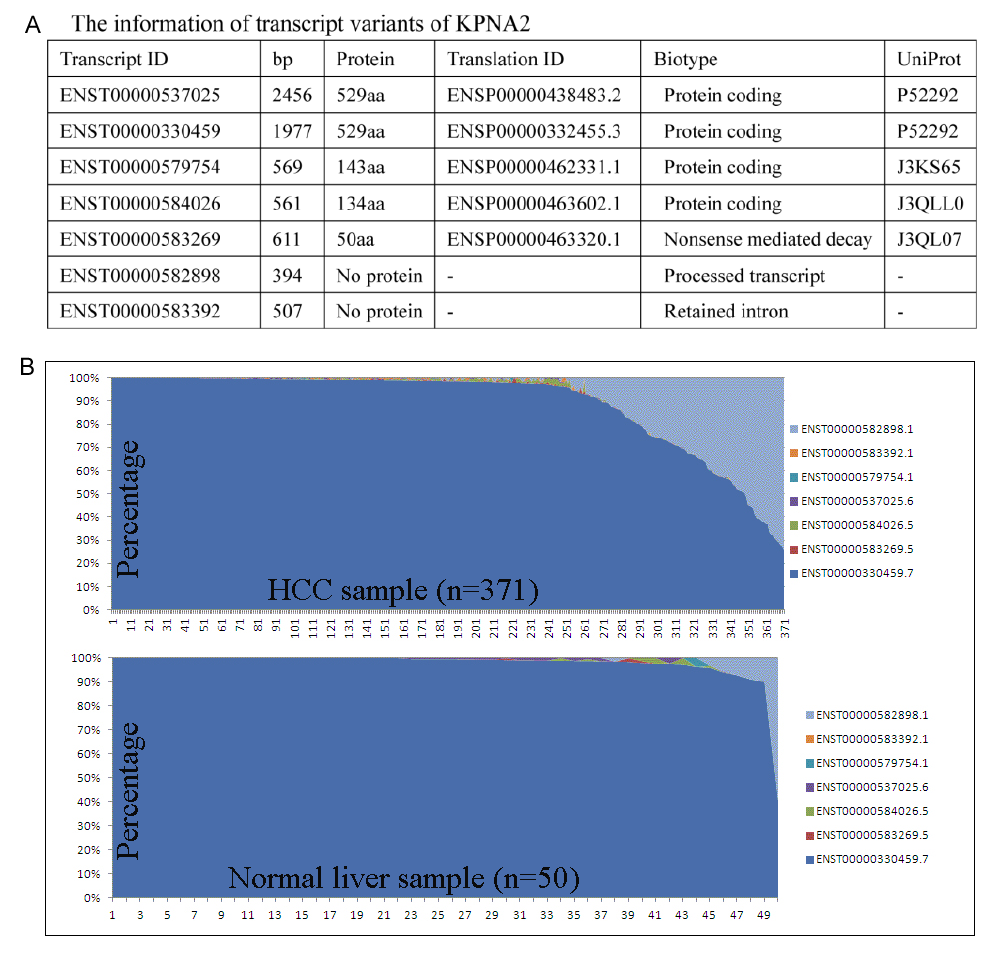
**

**Figure S6**. Transcript information of KPNA2 and their expressional proportions in HCC and normal liver tissues. (A) The transcript variants of KPNA2. (B) The expressional proportions of KPNA2 transcript variants in HCC and normal liver tissues. The transcript information of KPNA2 was investigated through ENSEMBL database (<https://www.ensembl.org/>). The 371 primary tumor samples and their paired 50 HCC patients were included for the evaluation of the expressional proportions of KPNA2 transcripts.
